# Crystal structure of a second polymorph of tricarbon­yl(*N*-methyl­pyridine-2-carboxamide-κ^2^ *N* ^1^,*O*)(thio­cyanato-κ*N*)rhenium(I)

**DOI:** 10.1107/S205698901601389X

**Published:** 2016-09-05

**Authors:** Krzysztof Lyczko

**Affiliations:** a Institute of Nuclear Chemistry and Technology, Dorodna 16, 03-195 Warsaw, Poland

**Keywords:** crystal structure, tri­carbonyl­rhenium(I) complex, *N*-methylpyridine-2-carb­oxy­amide ligand, thio­cyanate ion, polymorphism

## Abstract

A new polymorph of the title compound, [Re(NCS)(C_7_H_8_N_2_O)(CO)_3_], crystallizing in the space group *P*2_1_/*n*, has been obtained and structurally characterized by the experiment and DFT calculations. In this complex, the rhenium(I) cation is octa­hedrally coordinated by three carbonyl groups in a facial configuration, the *N*,*O*-bidentate *N*-methyl­pyridine-2-carboxamide ligand and the *N*-bonded thio­cyanate anion. Neighbouring mol­ecules are linked into a three-dimensional network by inter­molecular N—H⋯S and C—H⋯S inter­actions.

## Abstract 2

The second polymorph (monoclinic form) of the [Re(NCS){*L*H*(Me)*_NO_}(CO)_3_] complex, where LH*(Me)*_NO_ is *N*-methyl­pyridine-2-carboxamide, has been obtained and structurally characterized by X-ray diffraction and supported by DFT calculations.

## Chemical context   

Tri­carbonyl­rhenium(I) complexes in the ‘2 + 1’ system (with one bidentate and one monodentate ligand) are still widely studied because of their inter­esting photophysical and photochemical properties (Pizarro *et al.*, 2015 ▸; Zhao *et al.*, 2015 ▸; Portenkirchner *et al.*, 2015 ▸) and possible applications in medicine (Ma *et al.*, 2014 ▸; Wähler *et al.*, 2014 ▸; Collery *et al.*, 2015 ▸). Recently, a few tricarbonyl compounds of rhenium(I) with the bidentate *N*,*O*-donor ligand *N*-methylpyridine-2-carb­oxy­amide [*L*H*(Me)*_NO_] and with different monodentate ligands being either an anion (Cl^−^, Br^−^, I^−^ and SCN^−^) or a neutral mol­ecule [imidazole (Him) and 3,5-di­methyl­pyrazole (Hdmpz)] have been characterized, among others, by X-ray crystallographic analysis (Lyczko *et al.*, 2015 ▸). The first polymorph of the title complex [Re(CO)_3_(*L*H*(Me)*_NO_)NCS] to be reported (Lyczko *et al.*, 2015 ▸) has triclinic symmetry and crystallizes in the space group *P* 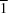.
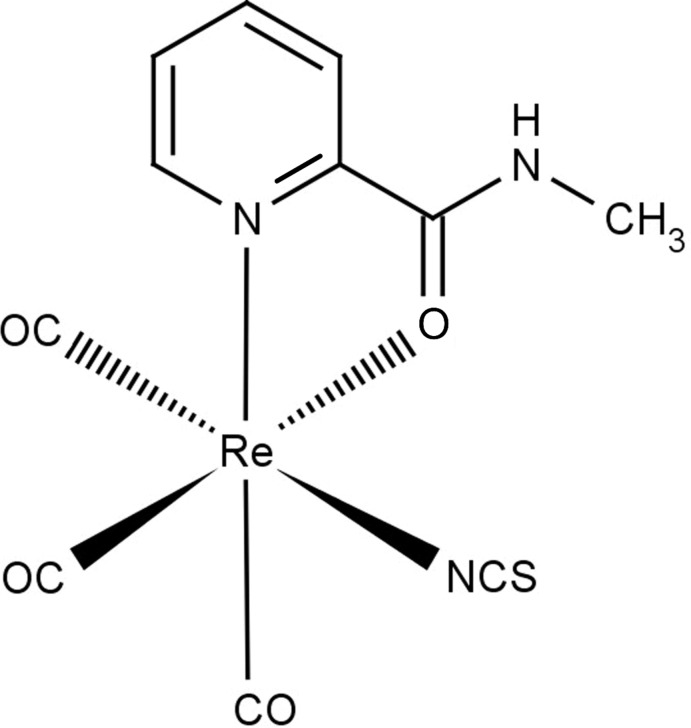


In the current study, a second polymorph of this compound crystallizing in the monoclinic space group *P*2_1_/*n* has been obtained and its structure is reported here, including a comparison of the triclinic and monoclinic polymorphs.

## Structural commentary   

**Figure 1:**
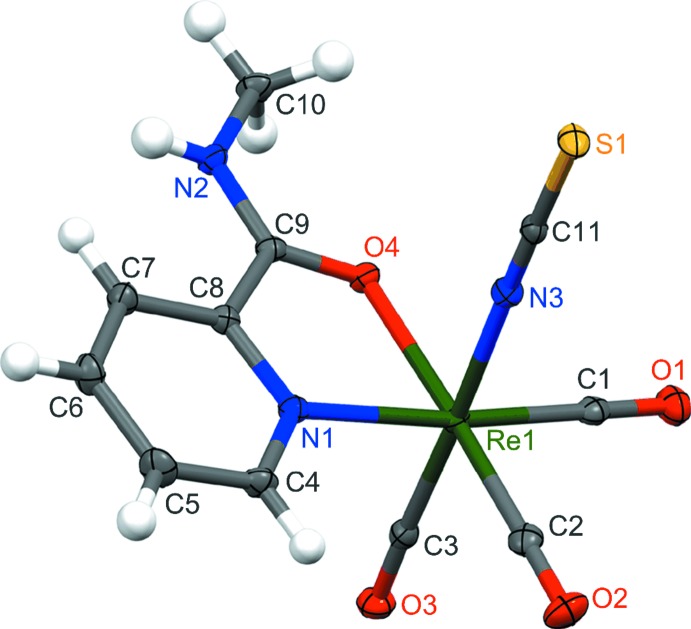
The mol­ecular structure of the title compound, with displacement ellipsoids for the non-H atoms drawn at the 50% probability level.

**Figure 2:**
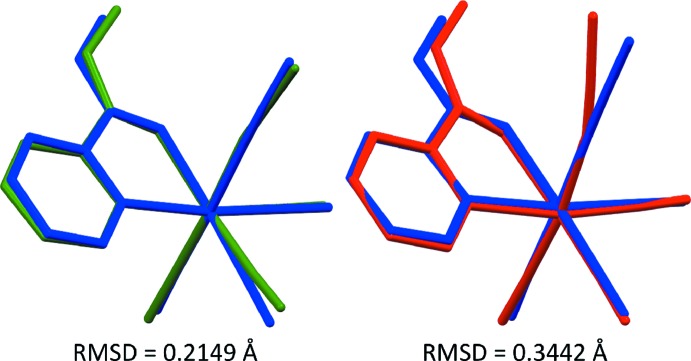
Graphical representations showing the overlays of the mol­ecular structures obtained for the [Re(CO)_3_(*L*H*(Me)*_NO_)NCS] complex from diffraction experiments and DFT calculations. The monoclinic form is blue, the triclinic form green and the DFT-optimized structure [B3LYP/LANL2DZ,6–311 G++(d,p)] is red.

The mol­ecular structure of the monoclinic polymorph of the studied tri­carbonyl­rhenium(I) complex with a bidentate ligand and a pseudohalide anion is presented in Fig. 1 ▸. The metal ion is surrounded in a slightly distorted octa­hedral coordination environment by six donor atoms, including three carbon atoms of the carbonyl groups, two nitro­gen atoms and one oxygen atom. The three CO ligands occupy the facial positions of this octa­hedron. The Re—C bond lengths are in the range 1.9028 (16)–1.9201 (16) Å. The three remaining positions in the *fac*-[Re(CO)_3_]^+^ core are occupied by one bidentate ligand and one monodentate ligand, which results in a so called ‘2 + 1’ system. *N*-methylpyridine-2-carboxy­amide behaves in the complex as a neutral ligand and chelates the rhenium(I) ion by means of oxygen and nitro­gen atoms with bond lengths of 2.1583 (10) and 2.1836 (13) Å, respectively, forming a five-membered ring. The N1—Re1—O4 bite angle of 74.33 (4)° is typical for that type of chelate ring. The sixth coordination position of the metal ion is occupied by the N atom of the thio­cyanate anion. The use of the NCS^−^ ion in the reaction mixture together with an *L*H*(Me)*_NO_ ligand leads to the formation of a neutral complex. This pseudohalide ion, which can exhibit an ambidentate character acting with the central metal cation either by its sulfur or nitro­gen atom, coordinates in the present complex through the N atom, which is generally typical for hard metal ions using the ‘hard and soft acids and bases’ (HSAB) concept. All of the structural parameters mentioned above are very similar to those previously reported for the triclinic polymorph of the title compound (see Table 1 ▸). The mol­ecular structures of the two polymorphic forms are compared in Fig. 2 ▸.

It can be ruled out that the use of AgBF_4_ for precipitation of Cl^−^ ions during the synthesis of the title complex (see Section 5) leads to the crystallization of its monoclinic polymorph, while the presence of PF_6_^−^ anions, originating from the silver salt, contributes to the formation of its triclinic form (Lyczko *et al.*, 2015 ▸).

## DFT calculations   

The bond lengths and angles for the present complex origin­ating from the crystal structure determination are in good agreement with DFT calculations (see Table 1 ▸) performed by means of the B3LYP functional and three different basis sets for non-metallic atoms (the Re atom was described by the LANL2DZ basis set) using the *GAUSSIAN09* software (Frisch *et al.*, 2009 ▸). In most cases, the differences between experimentally and theoretically determined atomic distances are no larger than 0.03 Å. In only a few cases, this difference larger than 0.03 Å, with the largest difference being about 0.08, 0.07 or 0.06 Å using the 6-31G(d,p), 6-31G++(d,p) or 6-311G++(d,p) basis sets, respectively, for the Re1—O4 bond length. The use of three different basis sets gave similar results. However, a slightly better correlation with the experiment can be observed by using the 6-311G++(d,p) basis set. It is especially noticeable if the C12—N3—Re1 angle and the bond lengths involving the chelating atoms (Re1—N1 and Re1—O4) are compared. The good agreement between the DFT-optimized and the experimentally determined structures is illustrated in Fig. 2 ▸.

## Supra­molecular features   

**Figure 3:**
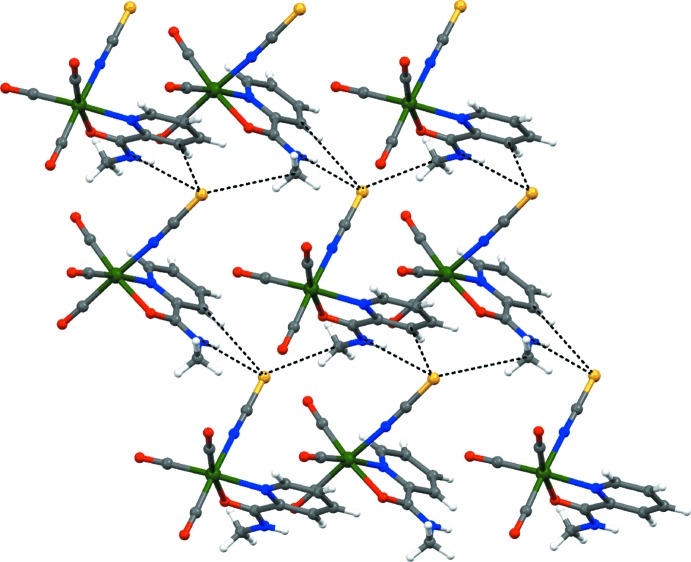
Fragment of the crystal structure of the title complex showing N—H⋯S and C—H⋯S hydrogen-bonding inter­actions as dashed lines.

The mol­ecular structure of both polymorphic forms of the [Re(CO)_3_(*L*H*(Me)*_NO_)NCS] complex are very similar, but their crystal structures display different packing features. In the crystal structure of the monoclinic polymorph, the mol­ecules are held together by N2—H2⋯S1 hydrogen bonds [3.3642 (14) Å] and two other weaker inter­actions [C7—H7⋯S1, 3.8255 (16) Å and C10—H10*C*⋯S1, 3.8445 (17) Å; Table 2 ▸, Fig. 3 ▸). In turn, the mol­ecular packing in the triclinic form is characterized by the presence of inter­molecular hydrogen bonds of 3.335 (3) Å (N2—H2⋯S1), 3.743 (4) Å (C6—H6⋯S1) and 3.921 (4) Å (C7—H7⋯S1) (Lyczko *et al.*, 2015 ▸). The shortest distances between neighbouring S atoms of the thio­cyanate ions [7.033 (1) and 7.175 (1) Å] in the monoclinic polymorph are much longer than the respective S⋯S contacts [4.736 (2) Å] in the triclinic form.

## Database survey   

The triclinic polymorph of the title complex has been presented recently (Lyczko *et al.*, 2015 ▸). Only a few crystal structures in which the thio­cyanate ion coordinates to a tri­carbonyl­rhenium(I) core can be found in the Cambridge Structural Database (Groom *et al.*, 2016 ▸) to date. In all these complexes, the thio­cyanato group inter­acts with the central metal atom in an *N*-bonded mode. The Re—N_(NCS)_ bond lengths in both polymorphs of [Re(CO)_3_(*L*H*(Me)*_NO_)NCS] [2.1275 (13) Å for the monoclinic form (this work) and 2.117 (3) Å for the triclinic form (Lyczko *et al.*, 2015 ▸)] are similar to other such bonds observed in [Re(CO)_3_(bipy(CH_3_)(COOH))NCS] [2.125 (3) Å, Cavigli *et al.*, 2016 ▸], [Re(CO)_3_(*t*Bu-DAB)NCS] [2.115 (1) Å; Grupp *et al.*, 2014 ▸], [Re(CO)_3_(bipy-PdTPP)NCS] [2.132 (9) Å; Schneider *et al.*, 2011 ▸], [Re(CO)_3_(Pr-DAB)NCS] [2.115 (7) Å; Rodríguez *et al.*, 2005 ▸], [Re(CO)_3_(bipy)NCS] [2.123 (4) and 2.129 (4) Å; Rodríguez *et al.*, 2005 ▸] or [Re(CO)_3_(NCS)_3_](NEt_4_)_2_ [2.112–2.145 (10) Å; Abram *et al.*, 1996 ▸].

## Synthesis and crystallization   

The title complex was synthesized by refluxing a methanol solution (5.0 ml) of Re(CO)_5_Cl (0.050 g, 0.138 mmol) with *N*-methylpyridine-2-carb­oxy­amide (0.30 g, 0.220 mmol) and KSCN (0.020 g, 0.206 mmol) after previous precipitation of AgCl by means of AgBF_4_ (0.027 g, 0.139 mmol), similar to the method described earlier (Lyczko *et al.*, 2015 ▸). The volume of this solution was decreased in a desiccator under reduced pressure. A yellow crystalline material was obtained after storing the solution for a few weeks in a refrigerator. Crystallization yield: 0.022 g (34.4%). Elemental analysis calculated for C_11_H_8_N_3_O_4_ReS: C, 28.44; H, 1.74; N, 9.05. Found: C, 28.33; H, 2.12; N, 9.18%. From the obtained material several crystals were checked crystallographically; the monoclinic form was entirely found.

## Refinement   

Crystal data, data collection and structure refinement details are summarized in Table 3 ▸. H atoms bonded to C atoms were inserted in calculated positions with C—H = 0.98 (meth­yl) or 0.95 Å (aromatic) and refined isotropically using a riding model with *U*_iso_(H) equal to 1.5*U*_eq_(C) or 1.2*U*_eq_(C) for methyl and aromatic H atoms, respectively. In turn, the H atom of the NH pair was located in a difference Fourier map and its position was freely refined.

## Supplementary Material

Crystal structure: contains datablock(s) I. DOI: 10.1107/S205698901601389X/wm5320sup1.cif

Structure factors: contains datablock(s) I. DOI: 10.1107/S205698901601389X/wm5320Isup2.hkl

CCDC reference: 1501544

Additional supporting information: crystallographic information; 3D view; checkCIF report

## Figures and Tables

**Table 1:** Comparison of selected bond lengths, distances (Å) and angles (°) between the experiments and calculations from three different basis sets for the studied complex^(*a*)^

	Triclinic^(*b*)^	Monoclinic	6–31G(d,p)	6–31G++(d,p)	6–311G++(d,p)
Re1—C1	1.915 (4)	1.9180 (16)	1.9328	1.9306	1.9329
Re1—C2	1.901 (4)	1.9028 (16)	1.9052	1.9016	1.9033
Re1—C3	1.923 (4)	1.9201 (16)	1.9342	1.9297	1.9330
Re1—N1	2.190 (3)	2.1836 (13)	2.2272	2.2079	2.2049
Re1—O4	2.159 (2)	2.1583 (10)	2.2412	2.2257	2.2169
Re1—N3	2.117 (3)	2.1275 (13)	2.1268	2.1076	2.0982
C9—O4	1.261 (4)	1.2581 (17)	1.2524	1.2798	1.2773
C9—N2	1.309 (5)	1.3182 (18)	1.3384	1.3388	1.3388
C10—N2	1.461 (5)	1.4610 (19)	1.4630	1.4708	1.4698
N1⋯O4	2.620 (4)	2.623 (2)	2.6616	2.6496	2.6401
N1—Re1—O4	74.09 (10)	74.33 (4)	73.12	73.40	73.32
N1—Re1—N3	83.91 (12)	83.15 (5)	81.16	81.40	81.12
O4—Re1—N3	81.68 (11)	82.40 (5)	79.06	78.84	79.73
C11—N3—Re1	167.0 (1)	174.4 (1)	157.29	161.39	167.84
N1—Re1—C1	170.93 (13)	172.55 (6)	169.97	169.90	169.65
O4—Re1—C2	168.57 (12)	171.84 (6)	170.77	170.63	170.91
N3—Re1—C3	174.90 (12)	177.96 (6)	171.84	171.84	172.19

Notes: ^(*a*)^DFT Calculations were carried out by means of *GAUSSIAN09* software (Frisch *et al.*, 2009 ▸) using the B3LYP functional and the LANL2DZ basis set for the Re atom; ^(*b*)^data from Lyczko *et al.* (2015 ▸).

**Table 2:** Hydrogen-bond geometry (Å, °)

*D*—H⋯*A*	*D*—H	H⋯*A*	*D*⋯*A*	*D*—H⋯*A*
N2—H2⋯S1^i^	0.84 (2)	2.57 (2)	3.3642 (14)	158.0 (19)
C7—H7⋯S1^i^	0.95	2.90	3.8255 (16)	166
C10—H10*C*⋯S1^ii^	0.98	2.98	3.8445 (17)	148

Symmetry codes: (i) 
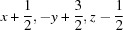
; (ii) 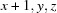.

**Table 3:** Experimental details

Crystal data	
Chemical formula	[Re(NCS)(C_7_H_8_N_2_O)(CO)_3_]
*M*_r_	464.46
Crystal system, space group	Monoclinic, *P*2_1_/*n*
Temperature (K)	100
*a*, *b*, *c* (Å)	8.3456 (1), 13.3241 (1), 12.7011 (1)
β (°)	99.284 (1)
*V* (Å^3^)	1393.83 (2)
*Z*	4
Radiation type	Mo *K*α
μ (mm^−1^)	8.88
Crystal size (mm)	0.15 × 0.12 × 0.08

Data collection	
Diffractometer	Agilent SuperNova Dual Source diffractometer with an Eos detector
Absorption correction	Multi-scan (*CrysAlis PRO*; Agilent, 2014 ▸)
*T*_min_, *T*_max_	0.629, 1.000
No. of measured, independent and observed [*I* > 2σ(*I*)] reflections	77774, 4060, 3921
*R*_int_	0.041
(sin θ/λ)_max_ (Å^−1^)	0.703

Refinement	
*R*[*F* ^2^ > 2σ(*F* ^2^)], *wR*(*F* ^2^), *S*	0.011, 0.025, 1.11
No. of reflections	4060
No. of parameters	186
H-atom treatment	H atoms treated by a mixture of independent and constrained refinement
Δρ_max_, Δρ_min_ (e Å^−3^)	0.42, −0.51

Computer programs: *CrysAlis PRO* (Agilent, 2014 ▸), *SHELXS97* (Sheldrick, 2008 ▸), *SHELXL2014* (Sheldrick, 2015 ▸), *Mercury* (Macrae *et al.*, 2008 ▸) and *publCIF* (Westrip, 2010 ▸).

## supplementary crystallographic information

### Crystal data

**Table:**

[Re(NCS)(C_7_H_8_N_2_O)(CO)_3_]	*F*(000) = 872
*M**_r_* = 464.46	*D*_x_ = 2.213 Mg m^−^^3^
Monoclinic, *P*2_1_/*n*	Mo *K*α radiation, λ = 0.71073 Å
*a* = 8.3456 (1) Å	Cell parameters from 49644 reflections
*b* = 13.3241 (1) Å	θ = 3.1–31.3°
*c* = 12.7011 (1) Å	µ = 8.88 mm^−^^1^
β = 99.284 (1)°	*T* = 100 K
*V* = 1393.83 (2) Å^3^	Block, yellow
*Z* = 4	0.15 × 0.12 × 0.08 mm

### Data collection

**Table:**

Agilent SuperNova Dual Source diffractometer with an Eos detector	4060 independent reflections
Radiation source: SuperNova (Mo) X-ray Source	3921 reflections with *I* > 2σ(*I*)
Detector resolution: 16.0131 pixels mm^-1^	*R*_int_ = 0.041
ω scans	θ_max_ = 30.0°, θ_min_ = 2.9°
Absorption correction: multi-scan (*CrysAlis PRO*; Agilent, 2014)	*h* = −11→11
*T*_min_ = 0.629, *T*_max_ = 1.000	*k* = −18→18
77774 measured reflections	*l* = −17→17

### Refinement

**Table:**

Refinement on *F*^2^	0 restraints
Least-squares matrix: full	Hydrogen site location: mixed
*R*[*F*^2^ > 2σ(*F*^2^)] = 0.011	H atoms treated by a mixture of independent and constrained refinement
*wR*(*F*^2^) = 0.025	*w* = 1/[σ^2^(*F*_o_^2^) + (0.0082*P*)^2^ + 0.8572*P*] where *P* = (*F*_o_^2^ + 2*F*_c_^2^)/3
*S* = 1.11	(Δ/σ)_max_ = 0.005
4060 reflections	Δρ_max_ = 0.42 e Å^−^^3^
186 parameters	Δρ_min_ = −0.51 e Å^−^^3^

### Special details

**Table:**

Geometry. All esds (except the esd in the dihedral angle between two l.s. planes) are estimated using the full covariance matrix. The cell esds are taken into account individually in the estimation of esds in distances, angles and torsion angles; correlations between esds in cell parameters are only used when they are defined by crystal symmetry. An approximate (isotropic) treatment of cell esds is used for estimating esds involving l.s. planes.

### Fractional atomic coordinates and isotropic or equivalent isotropic displacement parameters (Å^2^)

**Table:**

	*x*	*y*	*z*	*U*_iso_*/*U*_eq_	
Re1	0.94102 (2)	0.61839 (2)	0.78536 (2)	0.01041 (2)	
S1	0.39614 (5)	0.76937 (3)	0.72060 (3)	0.01731 (8)	
O2	0.81867 (15)	0.46596 (9)	0.93364 (10)	0.0228 (3)	
N2	0.96200 (16)	0.75572 (10)	0.48766 (10)	0.0133 (2)	
O1	1.07960 (14)	0.74704 (9)	0.97900 (9)	0.0203 (2)	
C2	0.86838 (19)	0.52386 (12)	0.87960 (12)	0.0161 (3)	
O4	0.99371 (13)	0.71917 (8)	0.66237 (8)	0.0127 (2)	
N1	0.85068 (15)	0.54369 (9)	0.63442 (10)	0.0125 (2)	
O3	1.27172 (14)	0.51162 (9)	0.82262 (9)	0.0184 (2)	
C3	1.14740 (19)	0.55136 (11)	0.80679 (11)	0.0135 (3)	
C1	1.02572 (18)	0.69876 (11)	0.90694 (12)	0.0145 (3)	
C5	0.7205 (2)	0.41062 (12)	0.52690 (14)	0.0192 (3)	
H5	0.6744	0.3453	0.5226	0.023*	
C6	0.7256 (2)	0.46596 (12)	0.43525 (13)	0.0184 (3)	
H6	0.6811	0.4396	0.3673	0.022*	
C8	0.85873 (18)	0.59693 (11)	0.54460 (12)	0.0120 (3)	
C4	0.78361 (19)	0.45171 (11)	0.62492 (13)	0.0159 (3)	
H4	0.7794	0.4136	0.6876	0.019*	
C9	0.94147 (17)	0.69584 (11)	0.56703 (11)	0.0116 (3)	
N3	0.71182 (16)	0.69184 (10)	0.75566 (10)	0.0149 (2)	
C7	0.79640 (19)	0.56049 (12)	0.44386 (12)	0.0157 (3)	
H7	0.8021	0.5995	0.3820	0.019*	
C10	1.0420 (2)	0.85309 (12)	0.50654 (13)	0.0166 (3)	
H10A	0.9883	0.8922	0.5562	0.025*	
H10B	1.0349	0.8894	0.4389	0.025*	
H10C	1.1563	0.8430	0.5372	0.025*	
C11	0.58131 (19)	0.72422 (11)	0.74029 (11)	0.0130 (3)	
H2	0.921 (3)	0.7413 (16)	0.4243 (18)	0.026 (6)*	

### Atomic displacement parameters (Å^2^)

**Table:**

	*U*^11^	*U*^22^	*U*^33^	*U*^12^	*U*^13^	*U*^23^
Re1	0.01001 (3)	0.01132 (3)	0.00967 (3)	0.00108 (2)	0.00092 (2)	0.00183 (2)
S1	0.01461 (18)	0.02173 (19)	0.01450 (17)	0.00703 (14)	−0.00096 (13)	−0.00273 (14)
O2	0.0222 (6)	0.0262 (6)	0.0202 (6)	−0.0021 (5)	0.0040 (5)	0.0091 (5)
N2	0.0159 (6)	0.0124 (6)	0.0111 (6)	−0.0027 (5)	0.0008 (5)	0.0007 (4)
O1	0.0199 (6)	0.0212 (6)	0.0190 (6)	0.0012 (5)	0.0009 (5)	−0.0047 (4)
C2	0.0129 (7)	0.0196 (7)	0.0149 (7)	0.0029 (6)	−0.0005 (5)	0.0008 (6)
O4	0.0134 (5)	0.0126 (5)	0.0117 (5)	−0.0016 (4)	0.0008 (4)	0.0012 (4)
N1	0.0118 (6)	0.0120 (6)	0.0137 (6)	0.0010 (4)	0.0023 (5)	0.0009 (4)
O3	0.0155 (5)	0.0195 (5)	0.0199 (5)	0.0041 (4)	0.0016 (4)	0.0027 (4)
C3	0.0154 (7)	0.0134 (6)	0.0115 (6)	−0.0018 (5)	0.0014 (5)	0.0008 (5)
C1	0.0118 (7)	0.0156 (7)	0.0161 (7)	0.0035 (5)	0.0027 (5)	0.0030 (5)
C5	0.0205 (8)	0.0145 (7)	0.0236 (8)	−0.0067 (6)	0.0061 (6)	−0.0028 (6)
C6	0.0195 (8)	0.0182 (7)	0.0180 (7)	−0.0049 (6)	0.0048 (6)	−0.0053 (6)
C8	0.0110 (7)	0.0118 (6)	0.0133 (6)	0.0000 (5)	0.0022 (5)	0.0005 (5)
C4	0.0171 (7)	0.0124 (7)	0.0189 (7)	−0.0015 (5)	0.0051 (6)	0.0024 (5)
C9	0.0099 (6)	0.0117 (6)	0.0131 (6)	0.0016 (5)	0.0014 (5)	0.0011 (5)
N3	0.0155 (6)	0.0155 (6)	0.0136 (6)	0.0012 (5)	0.0021 (5)	0.0003 (5)
C7	0.0179 (7)	0.0161 (7)	0.0134 (7)	−0.0021 (6)	0.0036 (6)	−0.0006 (5)
C10	0.0194 (8)	0.0128 (7)	0.0170 (7)	−0.0038 (6)	0.0012 (6)	0.0022 (5)
C11	0.0165 (7)	0.0121 (6)	0.0103 (6)	−0.0006 (5)	0.0011 (5)	−0.0010 (5)

### Geometric parameters (Å, º)

**Table:**

Re1—C2	1.9028 (16)	O3—C3	1.1533 (19)
Re1—C1	1.9180 (16)	C5—C4	1.384 (2)
Re1—C3	1.9201 (16)	C5—C6	1.384 (2)
Re1—N3	2.1275 (13)	C5—H5	0.9500
Re1—O4	2.1583 (10)	C6—C7	1.388 (2)
Re1—N1	2.1836 (13)	C6—H6	0.9500
S1—C11	1.6394 (16)	C8—C7	1.389 (2)
O2—C2	1.1533 (19)	C8—C9	1.494 (2)
N2—C9	1.3182 (18)	C4—H4	0.9500
N2—C10	1.4610 (19)	N3—C11	1.158 (2)
N2—H2	0.84 (2)	C7—H7	0.9500
O1—C1	1.1494 (19)	C10—H10A	0.9800
O4—C9	1.2581 (17)	C10—H10B	0.9800
N1—C4	1.3446 (19)	C10—H10C	0.9800
N1—C8	1.3542 (18)		
			
C2—Re1—C1	88.66 (7)	C4—C5—H5	120.4
C2—Re1—C3	88.42 (6)	C6—C5—H5	120.4
C1—Re1—C3	86.56 (6)	C5—C6—C7	119.17 (15)
C2—Re1—N3	92.72 (6)	C5—C6—H6	120.4
C1—Re1—N3	95.16 (6)	C7—C6—H6	120.4
C3—Re1—N3	177.96 (5)	N1—C8—C7	122.06 (14)
C2—Re1—O4	171.84 (5)	N1—C8—C9	112.69 (13)
C1—Re1—O4	98.26 (5)	C7—C8—C9	125.25 (13)
C3—Re1—O4	96.27 (5)	N1—C4—C5	122.25 (14)
N3—Re1—O4	82.40 (4)	N1—C4—H4	118.9
C2—Re1—N1	98.66 (6)	C5—C4—H4	118.9
C1—Re1—N1	172.54 (6)	O4—C9—N2	121.26 (14)
C3—Re1—N1	95.00 (5)	O4—C9—C8	118.64 (13)
N3—Re1—N1	83.15 (5)	N2—C9—C8	120.08 (13)
O4—Re1—N1	74.33 (4)	C11—N3—Re1	174.40 (12)
C9—N2—C10	121.62 (13)	C6—C7—C8	118.77 (14)
C9—N2—H2	120.5 (15)	C6—C7—H7	120.6
C10—N2—H2	117.6 (15)	C8—C7—H7	120.6
O2—C2—Re1	177.17 (14)	N2—C10—H10A	109.5
C9—O4—Re1	117.61 (9)	N2—C10—H10B	109.5
C4—N1—C8	118.59 (13)	H10A—C10—H10B	109.5
C4—N1—Re1	125.03 (10)	N2—C10—H10C	109.5
C8—N1—Re1	116.34 (10)	H10A—C10—H10C	109.5
O3—C3—Re1	178.12 (13)	H10B—C10—H10C	109.5
O1—C1—Re1	178.53 (13)	N3—C11—S1	179.00 (14)
C4—C5—C6	119.15 (15)		
			
C4—C5—C6—C7	1.2 (2)	C10—N2—C9—O4	−1.8 (2)
C4—N1—C8—C7	1.7 (2)	C10—N2—C9—C8	179.84 (13)
Re1—N1—C8—C7	−176.03 (12)	N1—C8—C9—O4	0.19 (19)
C4—N1—C8—C9	−177.88 (13)	C7—C8—C9—O4	−179.33 (14)
Re1—N1—C8—C9	4.44 (16)	N1—C8—C9—N2	178.59 (13)
C8—N1—C4—C5	−1.1 (2)	C7—C8—C9—N2	−0.9 (2)
Re1—N1—C4—C5	176.40 (12)	C5—C6—C7—C8	−0.6 (2)
C6—C5—C4—N1	−0.4 (2)	N1—C8—C7—C6	−0.8 (2)
Re1—O4—C9—N2	176.79 (11)	C9—C8—C7—C6	178.65 (14)
Re1—O4—C9—C8	−4.83 (17)		

### Hydrogen-bond geometry (Å, º)

**Table:**

*D*—H···*A*	*D*—H	H···*A*	*D*···*A*	*D*—H···*A*
N2—H2···S1^i^	0.84 (2)	2.57 (2)	3.3642 (14)	158.0 (19)
C7—H7···S1^i^	0.95	2.90	3.8255 (16)	166
C10—H10*C*···S1^ii^	0.98	2.98	3.8445 (17)	148

Symmetry codes: (i) *x*+1/2, −*y*+3/2, *z*−1/2; (ii) *x*+1, *y*, *z*.
